# The VEGF pathway and the AKT/mTOR/p70S6K1 signalling pathway in human epithelial ovarian cancer

**DOI:** 10.1038/sj.bjc.6604921

**Published:** 2009-02-24

**Authors:** X B Trinh, W A A Tjalma, P B Vermeulen, G Van den Eynden, I Van der Auwera, S J Van Laere, J Helleman, E M J J Berns, L Y Dirix, P A van Dam

**Affiliations:** 1 Translational Cancer Research Group Antwerp, St Augustinus Hospital, Antwerp, Belgium; 2 Department of Gynaecological Oncology, Antwerp University Hospital, Antwerp, Belgium; 3 Department of Medical Oncology, Erasmus MC/Josephine Nefkens Institute, Rotterdam, The Netherlands

**Keywords:** mTOR, VEGFR2, angiogenesis, autocrine, VEGF-A, ovarian cancer

## Abstract

Vascular endothelial growth factor (VEGF)-A inhibitors exhibit unseen high responses and toxicity in recurrent epithelial ovarian cancer suggesting an important role for the VEGF/VEGFR pathway. We studied the correlation of VEGF signalling and AKT/mTOR signalling. Using a tissue microarray of clinical samples (*N*=86), *tumour cell* immunohistochemical staining of AKT/mTOR downstream targets, pS6 and p4E-BP1, together with tumour cell staining of VEGF-A and pVEGFR2 were semi-quantified. A correlation was found between the marker for VEGFR2 activation (pVEGFR2) and a downstream target of AKT/mTOR signalling (pS6) (*R*=0.29; *P*=0.002). Additional gene expression analysis in an independent cDNA microarray dataset (*N*=24) showed a negative correlation (*R*=−0.73, *P*<0.0001) between the *RPS6* and the *VEGFR2* gene, which is consistent as the gene expression and phosphorylation of S6 is inversely regulated. An activated tumour cell VEGFR2/AKT/mTOR pathway was associated with increased incidence of ascites (*χ*^2^, *P*=0.002) and reduced overall survival of cisplatin–taxane-based patients with serous histology (*N*=32, log-rank test, *P*=0.04). These data propose that VEGF-A signalling acts on tumour cells as a stimulator of the AKT/mTOR pathway. Although VEGF-A inhibitors are classified as anti-angiogenic drugs, these data suggest that the working mechanism has an important additional modality of targeting the tumour cells directly.

In the Western world, ovarian cancer has the highest mortality rate among gynaecological malignancies (Jemal *et al*, 2008). There is an unmet need for novel targeted therapies that can improve current treatment outcomes (Kelland, 2005). In early clinical studies, remarkable activity of single agents targeting VEGF-A has been observed (Martin and Schilder, 2007). The working mechanism of these anti-VEGF-A inhibitors is believed to be inhibition of angiogenesis as VEGF-A is a critical and major step in angiogenesis (Ferrara, 2004, 2005). As single agent, bevacizumab, the monoclonal humanised antibody against VEGF-A, yielded response rates of 15.9 and 21.0% in refractory ovarian cancer patients in phase II trials and is currently the subject of phase III trials (Burger, 2007; Burger *et al*, 2007; Cannistra *et al*, 2007). In comparison, although it is difficult to compare different phase II trials, the observed response rate with single-agent bevacizumab was only 3.3% in metastatic colorectal cancer (Giantonio *et al*, 2007). In a metastatic renal cell cancer phase II trial, the response of single-agent high-dose bevacizumab was found to be 10.3%, whereas in metastatic breast cancer the total objective response rate was 6.7% (Cobleigh *et al*, 2003; Yang *et al*, 2003). In addition to its apparently different antitumoural effect, the highest incidence of gastrointestinal perforations (6%) is observed in ovarian cancer compared with the overall incidence of 1.7% in patients who receive bevacizumab for malignancies (Badgwell *et al*, 2008). In one phase II trial, the incidence was even found to be 11% (Cannistra *et al*, 2007).

The distinct response rates and toxicity observed indicate that VEGF-A signalling may have different mechanisms depending on the tumour type studied and also that there is a particular prominent role for VEGF-A in ovarian cancer. Therefore, further research of VEGF-A mechanisms in ovarian cancer is warranted. Several reports have identified the VEGFR2, also known as KDR, on epithelial tumour cells, including its localisation in ovarian cancer cells (Masood *et al*, 2001; Stewart *et al*, 2003; Inan *et al*, 2006). *In vitro* studies have suggested an autocrine growth factor function of VEGF-A/VEGFR2 signalling (Masood *et al*, 2001; Inan *et al*, 2006). However, the clinical importance of these non-endothelial VEGF receptors remains elusive. In this study, we assessed the VEGF-A/VEGFR2 pathway in human ovarian carcinoma tissue in terms of VEGF expression, VEGFR2 expression, and its activation status. As VEGFR2 exerts its function through the PI3K/Akt/mTOR signalling pathway in endothelial cells (Figure 1), the activation status of VEGFR2 was studied by determining phosphorylation of VEGFR2 itself together with phosphorylation of downstream targets of mTOR. In addition, the association of VEGFA, VEGFR2 and their activation status with clinical outcome were established.

## Materials and methods

### Patient and tissue selection

**Figure 2:**
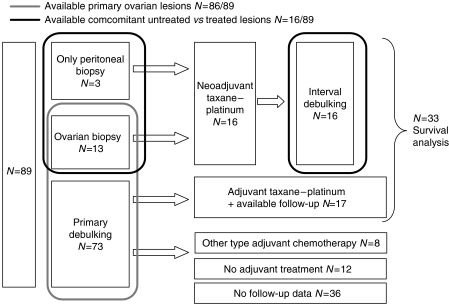
Schematic overview of patients included in the study.

Formalin-fixed paraffin-embedded (FFPE) epithelial ovarian cancer tissues were collected retrospectively from 1999 to 2004 in the St Augustinus hospital in Belgium using pathology archives. The local IRB reviewed and approved the study. Clinical parameters were obtained from patients’ medical files, that is, FIGO stage, grade, age at the time of diagnosis, date and type of surgery, histology, presence of ascites and treatment modalities. Remaining tumour load after debulking, which was not consistently recorded, was not included. The patients’ characteristics are listed in Table 1 and the biopsy characteristics in Figure 2. Median age of the patients was 61.6 years (27–92 years). Twelve patients did not receive systemic treatment. The majority received platinum in combination with taxane chemotherapy (*N*=33). Other treatments included carboplatinum monotherapy (*N*=1), carboplatinum–cyclofosfamide (*N*=5) and cyclophosphamide–adriamycin–cisplatin (*N*=2).

Tissue samples from 89 patients were included on the tissue microarray (TMA). Primary untreated ovarian lesions were available in 86 out of 89 patients, whereas in the remaining three subjects, only an untreated peritoneal biopsy was available (Figure 2). In 49 out of 89 patients, metastatic (peritoneal or omental) lesions were surgically removed at the same time by laparotomy, either during primary debulking or during interval debulking. Tissues were also collected from patients who had neoadjuvant chemotherapy before debulking surgery. In these patients, (16 out of 89) there was tissue from baseline biopsy and tissue from debulking surgery, which were all performed after three cycles of taxane–platinum chemotherapy.

### Tissue microarray

Using the Beecher Instruments Tissue Arrayer (Beecher Instruments, Silver Springs, MD, USA) a TMA was constructed. Haematoxylin eosin (HE) staining for each paraffin block was reviewed and three representative areas were selected on the HE slide. From each paraffin block, three sample cores were taken, each of which corresponded to the earlier selected areas on the HE slide. Five-*μ*m slides were cut from the TMA for immunohistochemical staining.

### Immunohistochemistry

Immunohistochemistry was performed on consecutive slides for pS6, p4E-BP1, pVEGFR2(Tyr996) (Cell Signalling Technologies, Beverly, MA, USA; #2211, #2474, #9451), pVEGFR2(Tyr951) (Santa Cruz Biotechnologies, Santa Cruz, CA, USA; #sc-16628-R) and VEGF-A (Dako Corp., Glostrup, Denmark, VG1clone M7273). After deparaffinisation and rehydration, antigen retrieval was performed using a citrate buffer (pH6.0) (pS6, p4E-BP1, VEGF-A) or an EDTA buffer (pH9.0) (pVEGFR2) at sub-boiling temperature for 30 min. After 30 min of cooling on bench top, sections were treated with 1% H_2_O_2_ and then incubated with the primary antibodies for 1 h at room temperature at 1 : 200 dilution for pS6, 1 : 50 for p4E-BP1, 1 : 100 for pVEGFR2(Tyr996) and 1 : 150 for pVEGFR2(Tyr951). Binding of antibodies was visualised using Envision*plus* dual link system (Dako Corp.) and 3,3-diaminobenzidine for 10 min. Sections were counterstained with Mayer's haematoxylin. For the VEGF-A staining, a 1 : 800 dilution with 1-h incubation was used with the Catalyzed Signal Amplification kit (CSA kit Dako Corp.). Ki67 staining was performed as described earlier (Van den Eynden *et al*, 2007). A breast carcinoma and melanoma (for VEGF-A, pVEGFR2), and a prostate cancer specimen (for pS6, p4E-BP1) served as positive controls. Negative controls were run by omitting the primary antibody. All the staining was done on the Dako autostainer (Dako Corp.).

### Quantifictation of the staining

The *H*-score, that is, the percentage of cells staining positive multiplied by an intensity score (0–3), was used for semi-quantification of the staining on the tumour cells (Rojo *et al*, 2007). The semi-quantification was performed by two independent observers who were blinded from clinical data. As three cores were taken from a single paraffin block, the average *H*-score over the three cores was used for further analysis. Ki67 staining was quantified with a proliferation index (number of Ki67 positive proliferating tumour cells per 100 tumour cells) using 200 counted cells per tissue core.

### Statistical and survival analysis

All statistical analyses were performed using SPSS 13.0 (SPSS Inc., Chicago, IL, USA) and GraphPad Prism 3.03 (GraphPad Software, Inc., La Jolla, CA, USA) statistical packages. Marker correlation analysis of *H*-scores was performed using the Spearman correlation coefficient. For survival analysis, only those patients who had taxane–platinum first line chemotherapy and serous papillary histology were selected (*N*=32). For this, the expression of pVEGFR2 and pS6 was dichotomised in high or low category using the median expression as a cutoff value. Wilcoxon matched paired testing was performed on the *H*-scores if primary ovarian tissue as well as a metastatic lesion was available from the same patient, at the same time of surgical excision (*N*=49). Similarly, Wilcoxon matched paired testing was conducted if the tissue, before chemotherapeutical treatment and after neoadjuvant taxane–platinum chemotherapy, was available from the same patient (*N*=16).

### *In silico* gene expression analysis

Normalised gene expression data was derived from a molecular profiling study described earlier, including 24 independent untreated primary ovarian cancer lesions, using 18K cDNA microarray (Helleman *et al*, 2006; Erasmus MC, Rotterdam, The Netherlands). The expression values of *RPS6* (coding for S6 protein), *EIF4EBP1* (coding for 4E-BP1 protein) and *VEGFR2/KDR* were analysed for correlation studies. The mean of duplicate analyses was used. In addition, gene expressions for *RPS6* and *EIF4EBP1* were derived from a publicly available gene expression omnibus dataset of *Mus musculus* prostate samples before and after (12 and 48 h) mTOR inhibition with the RAD001 compound. These samples were processed using Affymetrix GeneChip Mouse Expression Set 430 Array MOE430A (Affymetrix Inc., Santa Clara, CA, USA). Microarrays were background adjusted, normalised, summarised and ^2^log transformed according to GC Robust Miroarray method. Nine probe set ID's were available for analysis of the *RPS6* gene and two were available for *EIF4EBP1* gene. Samples were divided into three groups: placebo treated (*N*=18), 12 h RAD001 treated (*N*=11) and 48 h RAD001 treated (*N*=9). One-way ANOVA tests with additional Student–Newman–Keul's post test (if *P*<0.05) were performed.

## Results

### Immunostaining and marker correlative studies

**Figure 3:**
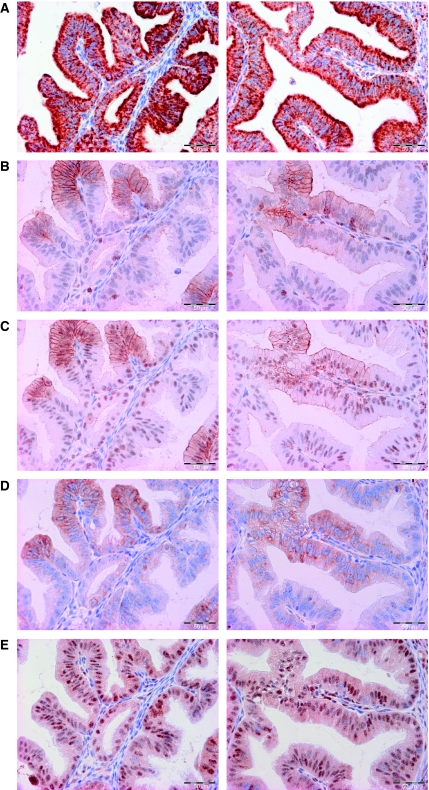
(**A**–**E**) Serial slides with immunohistochemical staining for pVEGFR2(Tyr951) (**A**), pVEGFR2(Tyr996) (**B**), pS6 (**C**), p4E-BP1 (**D**), VEGF-A (**E**) of an ovarian cancer specimen. Note that there were locally places in which there was more prominent staining for pVEGFR2 with a concomitant expression of the ribosomal protein pS6.

**Figure 4:**
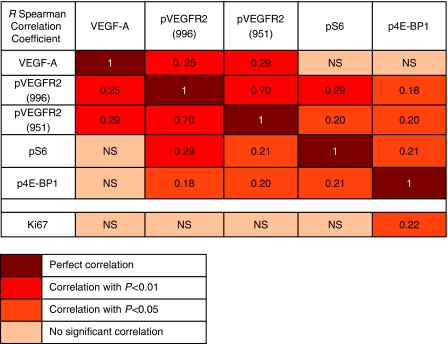
A summary of immunohistochemical correlations.

An example of staining of pVEGFR2(Tyr996), pVEGFR2(Tyr951) pS6, p4E-BP1 and VEGF-A is shown in Figure 3. A summary of the staining *H*-scores is listed in Table 1, whereas the correlations are presented in Figure 4. VEGF-A expression is most prevalent in serous papillary tumours (*P*=0.0002). The staining of the VEGFR2 phosphorylated at tyrosine residue 996 or 951 was found to be cytoplasmic, nuclear and/or membranous. There was a correlation (*R*=0.70, *P*<0.00001) between both pVEGFR2 staining, namely the VEGFR2 phosphorylated at tyrosine residue 951 and that phosphorylated at tyrosine residue 996, using different antibodies that were raised to detect different epitopes. The staining patterns for pS6, p4E-BP1 and nuclear ki67 staining were similar to the reports described earlier (Van den Eynden *et al*, 2005; Castellvi *et al*, 2006; Rojo *et al*, 2007). A predominantly often granular staining of VEGF-A was observed in the tumour cell cytoplasm as well in the stromal compartment of the tumour.

For correlations, (Figure 4) primary untreated ovarian lesions (*N*=86) were included. Correlations between pVEGFR2(Tyr996) and downstream markers for the AKT/mTOR signalling pathway (pS6 and p4E-BP1) were found for pVEGFR2(Tyr996) and pS6 (*R*=0.29, *P*=0.002), and also for pVEGFR2(Tyr996) and p4E-BP1 (*R*=0.18, *P*=0.05).

When studying only metastatic lesions (*N*=59), the correlation of pVEGFR2(Tyr996)–pS6 was even more pronounced (*R*=0.44, *P*<0.0001), whereas there was no correlation between pVEGFR2(Tyr996)–p4E-BP1 (*R*=0.12 *P*=0.25). Interestingly, the expression of pVEGFR2(Tyr996) was found to be significantly lower in metastatic lesions when compared with primary ovarian lesions surgically removed at the same time (median *H*-score=90, range (0–300) *vs* 150, range (0–300); *P*=0.01, *N*=49). The expression of pS6 and p4E-BP1 was not significantly different.

The tissue microarray also contained samples pre- and post-neoadjuvant chemotherapy. Although the study was retrospective, the administration of the neoadjuvant chemotherapy was according to hospital protocol and uniform; after three cycles of taxane–platinum chemotherapy an interval debulking surgery procedure was performed. Sixteen patients were eligible for analysis as pre- and post chemotherapy FFPE tissues were available (Figure 2). pVEGFR2(996) expression levels after chemotherapy were lower compared with the initial untreated biopsy (median *H*-score=50, range (0–300) *vs* 300, range (120–300); *P*<0.0001). For the pS6 and p4E-BP1 expression there was no significant difference found. Again, the correlation of pVEGFR2(Tyr996) and pS6, and pVEGFR2(Tyr996) and p4E-BP1 was present in lesions treated after chemotherapy (*R*=0.44, *P*=0.002–*R*=0.27 *P*=0.054).

### Gene expression analysis

**Figure 5:**
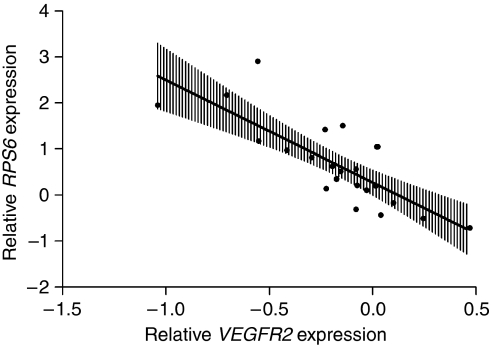
The ^2^log relative gene expression correlations using an independent dataset of epithelial ovarian cancer samples. The *RPS6* gene was significantly well correlated with the relative expression of *VEGFR2*. (vertical bars show 95% CI).

Next, the observed immunohistochemical correlation of pVEGFR2 and pS6 was studied *in silico* at a gene expression (mRNA) level from cDNA microarrays of 24 ovarian cancers from the Erasmus MC centre (Helleman *et al*, 2006). Relative gene expressions were correlated between the *VEGFR2* gene and the *RPS6* and *EIF4EBP1* genes. There was a highly significant, but negative, correlation between the *VEGFR2* and *RPS6* (*R*=−0.73; *P*<0.0001), whereas there was no correlation with *EIF4EBP1* (Figure 5). This negative correlation is compatible with the findings that the gene expression of S6 and its phosphorylation status is inversely regulated.

**Figure 6:**
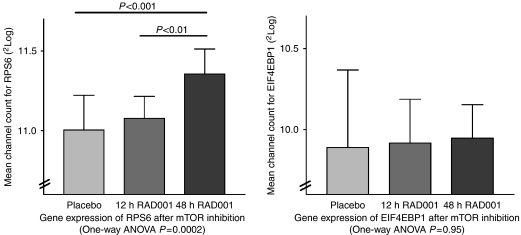
After 48 h RAD001 administration, prostate tissue showed a significant increase of normalised gene expression for *RPS6* compared with 12 h (*P*<0.01—^2^log mean channel count increase of +0.28 95% CI (0.08–0.48)) and placebo-treated mice (*P*<0.001—^2^log mean channel count increase of +0.35 95% CI (0.17–0.53)), This effect was not observed for the expression of *EIF4EBP1* (n.s.).

Phosphorylation of the ribosomal protein S6 is completely inhibited after mTOR inhibition, thus protein expression of pS6 is expected to decrease (Tabernero *et al*, 2005). *In silico* analyses (Affymetrix microarray data from prostate of *Mus musculus* treated with mTOR inhibitor RAD001) show that this downstream marker of the AKT/mTOR signalling pathway is upregulated after mTOR inhibition. A significant, apparently time dependant, increased gene expression after mTOR inhibition of the gene *RPS6* could be seen, whereas there was no significant change for *EIF4EBP1* (Figure 6).

### Survival analysis

**Figure 7:**
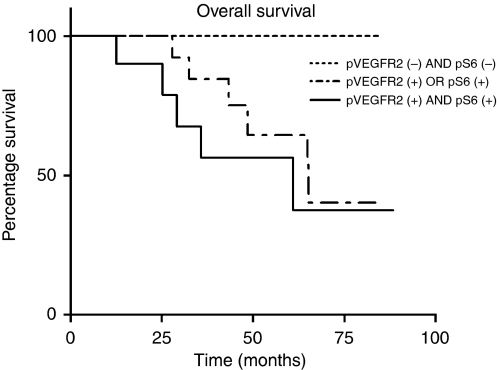
Kaplan–Meier survival curve (*N*=32 patients). Baseline expression of pS6 and pVEGFR2 were dichotomised into a high (+) and low (−) category using the median expression *H*-score value (log-rank test, *P*=0.041).

The expression of pS6 and VEGFR2(Tyr996) was dichotomised using the median expression as a cutoff value. Patients were divided into three groups: (1) patients with a high expression of pS6 and pVEGFR2(Tyr996) (*N*=8), ‘activated pathway’, (2) patients with a combined low expression of pS6 and pVEGFR2 in the tumour (*N*=9) and (3) the intermediate group, in which only one out of two markers had high expression (*N*=15). A significant reduced survival was found in patients who had a combined high expression of pVEGFR(Tyr996) and pS6 (log-rank test, *P*=0.041) (Figure 7). In an exploratory multivariate analysis using Cox Proportional Hazard regression model correcting for FIGO stage and grade, this significance is retained (*P*=0.048, HR 2.90, 95% CI (1.01–7.97)) and FIGO staging was also found to be a independent prognostic factor (*P*=0.050, HR 1.99, 95% CI (0.89–4.47)).

A combined high expression of pVEGFR2(Tyr996) and pS6 was significantly associated with the presence of ascites (87.5%, *N*=7/8), whereas in patients with a low expression of both markers, the presence of ascites was only 11.1% (*N*=1/9). In the intermediate group there was ascites in 40.0% (*N*=6/15) (*P*=0.002; *χ*^2^ test for linear by linear association).

## Discussion

Using human ovarian cancer samples, a correlation was observed between the activated status of the VEGFR2 and downstream markers of the AKT/mTOR signalling pathway, pS6 and p4E-BP1 protein. Especially, the correlation of pVEGFR2(Tyr996) and pS6 was found to be the most noteworthy correlation between VEGF-A signalling and AKT/mTOR signalling. For these two markers, besides the *H*-score correlations that were observed, a correlation could also be seen in heterogenic staining areas of some tumour samples, that is, places where pVEGFR2-positive cells also clearly stained more positive for pS6 (Figure 3). Two antibodies were used in this study for pVEGFR2. Mainly, the results of pVEGFR(Tyr996) were reported because pVEGFR2(Tyr996) seems to be a better potential marker for bevacizumab efficacy (Wedam *et al*, 2006). Similarly, for markers of the AKT/mTOR signalling pathway two antibodies were selected also, with a preference for pS6, as there is evidence that S6 is more specifically regulated by mTOR (Tabernero *et al*, 2008). However, p4E-BP1 was also chosen because it has been shown to be a hallmark protein downstream of mTOR that is associated with grade and survival in ovarian cancer as well as in breast cancer (Castellvi *et al*, 2006; Rojo *et al*, 2007).

Gene expression analysis showed that, after mTOR inhibition, the gene expression of S6 changed, whereas for 4E-BP1 it did not. Although the change fold (<2) was minimal, it was an apparently time dependant, clearly significant change. Here, the negative correlation of *VEGFR2* and *RPS6*, is compatible with the finding that the phosphorylation of S6 is inversely regulated with its gene expression. These data are therefore indirectly confirmative for the immunohistochemical findings that showed a positive correlation.

Given our current knowledge, there are many uncertainties and it is too early to rely completely on biomarkers for clinical usage. To fully appreciate the value of biomarkers, extensive translational research in considerable number of patients, are needed (Sleijfer and Wiemer, 2008).

Nevertheless, our findings strongly suggest activation of the VEGFR2 pathway in ovarian cancer. Consistent with our data, earlier, a similar study with human samples has shown the expression of the VEGFR2 to be been associated with the activation of the signal transducers and activators of transcription pathway, another intracellular signalling pathway of VEGFR2 (Chen *et al*, 2004). The most likely way by which this receptor is activated is through binding of its ligand, VEGF-A. Platelets are a major source of VEGF-A that is targeted by bevacizumab (Verheul *et al*, 2007). As the ovarian tumour cells are known to excrete the endothelial growth factor VEGF-A as well, this suggests an autocrine/paracrine growth factor function of VEGF-A in ovarian cancer through the AKT/mTOR signalling pathway. The dual expression of VEGFR/VEGF-A in ovarian cancer, as well as in other types of cancers, suggests that the activation of tumour cell signalling pathways by VEGF-A might be an autocrine mechanism that could also be present in other tumours such as colorectal or breast carcinoma (Masood *et al*, 2001; Price *et al*, 2001; Chen *et al*, 2004; Fan *et al*, 2005; Wedam *et al*, 2006).

Anti-angiogenesis with agents such as bevacizumab are believed to act through blocking VEGF-A action on endothelial cells. The unseen antitumoural effects observed after bevacizumab treatment in refractory- and platinum-resistant ovarian cancer patients indicate that these responses possibly are not only caused by inhibition of angiogenesis. We hereby show that a presumed activated VEGFR2/AKT/mTOR pathway, that is *endothelial cell independent*, was found to be associated with significantly reduced survival. Although the study cohort was small, without searching for a cutoff value, significance could be shown. The fact that this activated pathway was associated with increasing incidence of ascites is maybe not surprising. VEGF-A, formerly know as vascular permeability factor, has been found to play an important role in the accumulation of pleural or peritoneal effusions (Senger *et al*, 1983; Nagy *et al*, 1993, 1995; Yeo *et al*, 1993; Masood *et al*, 2001). Also, in preclinical studies, the bevacizumab murine precursor was more an inhibitor of ascites formation than that it was an inhibitor of tumour growth (Mesiano *et al*, 1998).

The observed correlation between the VEGF-A pathway and the AKT/mTOR pathway has potential clinical importance. mTOR inhibitors gain much clinical interest as an antitumoural agent, such as in renal cell carcinoma (Hudes *et al*, 2007). For ovarian cancer, clinical trials are ongoing. Up to date, there is only preclinical data available to suggest that mTOR inhibition might be beneficial in epithelial ovarian cancer. Altomare *et al*, 2004 studied the phosphorylation of AKT on ovarian cancers in a tissue microarray and found overexpression of p-AKT on 68% of the 31 tumours. Mabuchi *et al*, 2007a, 2007b found that mTOR inhibition by RAD001 reduced human ovarian cancer cell proliferation, enhanced cisplatin-induced apoptosis and prolonged survival in an ovarian cancer xenograft model. They also showed that RAD001 inhibited the expression of HIF 1 alpha and VEGF-A *in vitro* cell lines. Interestingly, dual targeting of VEGF-A and mTOR in ovarian caner xenograft models has shown an additive, if not synergistic, antitumoural effect with survival benefit. Additionally, the combination therapy was able to reverse the accumulation of ascites, which is in agreement with our findings (Huynh *et al*, 2007).

Anti-VEGF treatments in ovarian cancer seem to be very active, although at this moment, the associated toxicity is worrisome. mTOR inhibitors might have the potential of avoiding these problems Taking our data into consideration, suggestive of an autocrine VEGF-A loop through the AKT/mTOR signalling pathway, this adds preclinical rationale for mTOR inhibition in the management of ovarian cancer. The results of the GOG phase II trial, which is ongoing, will reveal if temsirolimus has single-agent activity in recurrent/refractory patients.

We started a multicentre prospective study in 2006 with the aim of standardised collection of snap frozen human ovarian cancer tissues. Similar experiments will reveal if our present findings can be confirmed. We will try to further elucidate the interaction between both the pathways at a more detailed gene expression level. In any future clinical trials, we emphasise the necessity of tissue/ascites sampling for translational and biomarker studies.

In conclusion, we propose that the working mechanism of anti-VEGF treatments in epithelial ovarian cancer is not only anti-angiogenesis. We strongly suggest that these anti-VEGF treatments are suppressors of epithelial tumour cell growth factor acting as a surrogate AKT/mTOR signalling inhibitors on tumour cells. Thus, classifying VEGF trap or bevacizumab as anti-angiogenic agent does not represent their whole mechanism of action. Based on our findings, we recommend them as anti-VEGF compounds, at least in epithelial ovarian cancer.

## Figures and Tables

**Figure 1:**
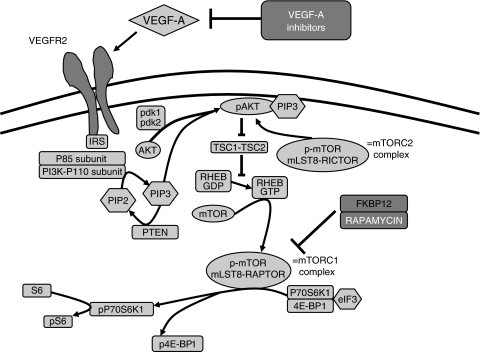
In endothelial cells, after activation, VEGFR2 is able to stimulate the AKT/mTOR signalling pathway through PI3K. More downstream 4E-BP1, P70S6K1 and S6 are phosphorylated. The mTORC2 complex (insensitive to rapamycin/FKBP12) has been shown to be an activator of AKT. The mTORC1 complex is sensitive to rapamycin (sirolimus) or derivates (everolimus, temsirolimus and deforolimus).

**Table 1:** Summary of clinical data (*N*=89 patients)

	***N***	**pVEGFR2(Tyr996)**	**pVEGFR2(Tyr951)**	**pS6**	**p4E-BP1**	**VEGF-A**	**Ki67**
FIGO I	19	270	275	20	100	200	18.5
FIGO II	4	285	100	45	125	150	20.0
FIGO III	56	210	100	20	90	200	19.0
FIGO IV	10	300	180	150	160	200	18.0
		*NS*	*NS*	*NS*	*NS*	*NS*	*NS*
Grade 1	20	300	200	30	90	150	18.5
Grade 2	27	180	80	15	80	200	12.0
Grade 3	42	300	100	20	160	200	21.0
		*P*=*0.06*	*NS*	*NS*	*P*=*0.085*	*NS*	*NS*
Serous papillary	47	270	180	20	80	200	17.0
Mucinous	4	195	250	20	95	150	29.0
Endometrioid	23	210	80	25	160	100	23.5
Clear cell	10	195	50	40	95	100	4.5
		*NS*	*NS*	*NS*	*NS*	*P*=*0.0002*	*P*=*0.095*
Total	89	240 (0–300)	160 (0–300)	20 (0–300)	100 (2–300)	200 (0–300)	19.0 (0–66)

Median *H*-scores are reported. *P*-values (Kruskal–Wallis tests) are reported in case of significance (*P*<0.05) or in case of trend (*P*<0.10). After Bonferroni correction for multiple testing *P*<0.008 was considered significant.
